# The Actin Regulators Involved in the Function and Related Diseases of Lymphocytes

**DOI:** 10.3389/fimmu.2022.799309

**Published:** 2022-03-16

**Authors:** Jianxuan Sun, Xingyu Zhong, Xiaoyu Fu, Heather Miller, Pamela Lee, Bing Yu, Chaohong Liu

**Affiliations:** ^1^ Department of Pathogen Biology, School of Basic Medicine, Tongji Medical College, Huazhong University of Science and Technology, Wuhan, China; ^2^ Department and Institute of Urology, Tongji Hospital, Tongji Medical College, Huazhong University of Science and Technology, Wuhan, China; ^3^ Cytek Biosciences, R&D Clinical Reagents, Fremont, CA, United States; ^4^ Department of Paediatrics and Adolescent Medicine, Li Ka Shing Faculty of Medicine, The University of Hong Kong, Hong Kong, Hong Kong SAR, China

**Keywords:** actin regulators, T cell, B cell, NK cell, WAS

## Abstract

Actin is an important cytoskeletal protein involved in signal transduction, cell structure and motility. Actin regulators include actin-monomer-binding proteins, Wiskott-Aldrich syndrome (WAS) family of proteins, nucleation proteins, actin filament polymerases and severing proteins. This group of proteins regulate the dynamic changes in actin assembly/disassembly, thus playing an important role in cell motility, intracellular transport, cell division and other basic cellular activities. Lymphocytes are important components of the human immune system, consisting of T-lymphocytes (T cells), B-lymphocytes (B cells) and natural killer cells (NK cells). Lymphocytes are indispensable for both innate and adaptive immunity and cannot function normally without various actin regulators. In this review, we first briefly introduce the structure and fundamental functions of a variety of well-known and newly discovered actin regulators, then we highlight the role of actin regulators in T cell, B cell and NK cell, and finally provide a landscape of various diseases associated with them. This review provides new directions in exploring actin regulators and promotes more precise and effective treatments for related diseases.

## 1 Introduction

Actin is a highly conserved protein that is abundantly expressed in most eukaryotic cells. Like intermediate filaments and microtubules, actin is a major component of the cytoskeleton and plays an extremely significant role in the structure, motility, activation and maintenance of cells.

Actin exists in the form of monomers (globular actin or G-actin) or filamentous polymers (filamentous actin or F-actin). G-actin is formed by a polypeptide chain containing 375 amino acid residues, which can be structurally divided into two parts: the endostructural domain and the exostructural domain (1). There are two clefts between these two domains, the nucleotide-binding cleft and the target-binding cleft, which binds to nucleotides and proteins, respectively, to regulate the activity of actin. F-actin is a right-handed helical structure consisting of two chains coiled around each other (2). G-actin and F-actin can interconvert through polymerization and depolymerization, and this process often requires the assistance of several regulatory proteins and the involvement of various small molecules. ATP-bound G-actin has greater affinity and nucleation when interacting with nucleation proteins, such as the Arp2/3 complex, which form stable oligomers consisting of three to four monomers (3) that are then extended and assembled into F-actin. Simultaneously, F-actin undergoes depolymerization *via* the hydrolysis of ATP. The rate of actin polymerization and depolymerization depends on the available concentration of G-actin and ATP. Due to the polarity of F-actin, in order to extend a filament in one direction, the rate of polymerization at the one end (barbed end), must be relatively faster than the depolymerization at the other end (pointed end). This phenomenon, known as “treadmilling” (4), maintains the dynamic equilibrium between G-actin and F-actin.

As an important component of microfilaments, actin plays a critical role in the maintenance of cell structure (5). Under the action of “treadmilling”, filaments extend at barbed ends, forming protrusions at the leading edge of the cell (6). This is the basis for the formation of lamellipodia, microvilli and filopodia (7), which are necessary for cell motility and food intake. Together with other molecules, actin is also involved in cellular endocytosis (8). In addition, actin also interacts with myosin. Myosin has ATPase activity and hydrolyzes ATP to generate energy, leading to the sliding of actin and myosin filaments against each other, thus generating the tension required for the basic mechanism of muscle contraction (9). Similar processes are also present in non-muscle cells, and the resulting contractile force is important for cell migration, cytoplasmic division, or other biological processes.

**Figure 1:**
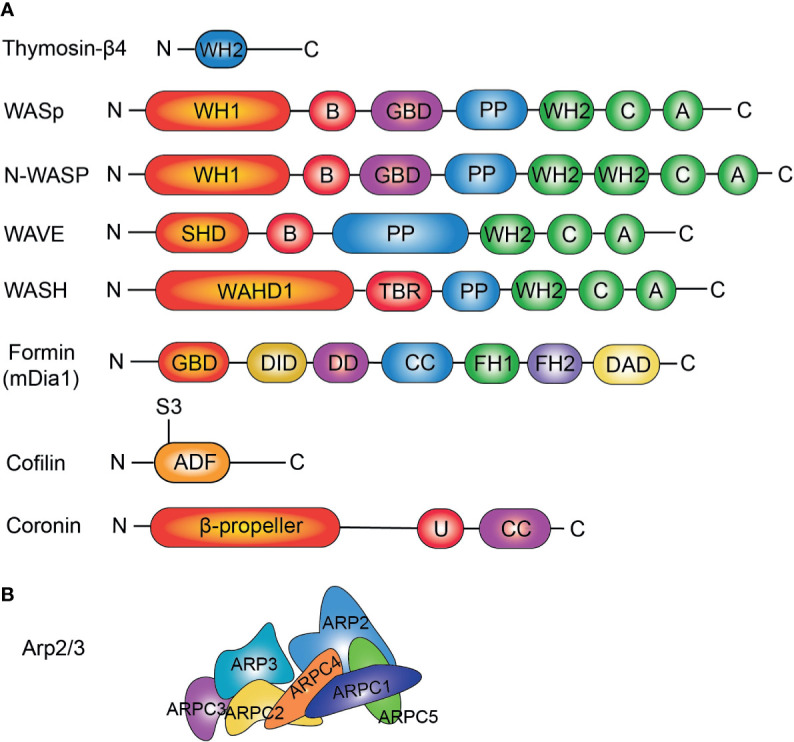
**(A)** Domain structures of actin regulators important for lymphocytes. THY, thymosin β actin-binding motif; WH1, WASP Homology domain-1; B, Basic domain; GBD, GTPase binding domain; PP, poly-proline; WH2, WASP Homology domain-2; C, Connecting sequence; A, Acidic sequence; SHD, SCAR-homology domain; WAHD1, WASH Homology domain; TBR, Tubulin binding region; HD, Helical domain; DID, diaphanous autoregulatory domain; DD, dimerization domain; CC, coiled coil region; FH1, formin homology domain 1; FH2, formin homology domain 2; DAD, diaphanous autoregulatory domain; ADF, actin depolymerizing factor; U, unique region; CC, coiled-coil. **(B)** Conformation of Arp2/3 complex. ARP2; ARP3; and ARP complex-1~5 (ARPC1~5) are shown.

Actin regulators are a series of proteins that control the assembly/disassembly dynamics of actin for cell motility, intracellular transport, muscle contraction, cellular structure maintenance, cytokinesis and other fundamental cell activities. Well studied actin regulators include: 1) Actin Capping Proteins that interact with actin and regulate the capping of the ends of actin polymers, 2) Actin Depolymerizing Factors that severe and depolymerize actin filaments, 3) Actin-Related Protein 2/3 Complex (Arp2/3) that nucleates branched actin, and 4) Wiskott-Aldrich Syndrome Protein (WASP) Family that interacts with and activates Arp2/3 (**Figure 1**) (10).

Lymphocytes are an important constituent of leukocytes in humans, and include T lymphocytes (T cells), B lymphocytes (B cells) and natural killer cells (NK cells) that play an essential role in both innate and adaptive immunity (11). T lymphocytes are divided into T helper cells (Th cells), cytotoxic T cells (CTLs), regulatory T cells (Tregs) and other subsets according to biomarkers on the cell membrane and their functions, which are mainly involved in cell-mediated immunity. However, they are also indispensable in humoral immunity *via* their helper effector functions, such as cytokines release. Whereas B lymphocytes are the major players of humoral immunity and the exclusive source of antibodies (12, 13), NK cells are crucial to the innate immune system, which function in a cell-mediated and cytotoxic way that is essential in defending against tumors and viral infections (13). These three types of lymphocytes help form a sophisticated immune network to recognize and eliminate non-self antigens, thus maintaining the stability of the internal environment in humans.

In lymphocytes, actin is of great significance for cell activation, adhesion, and migration (14–18). For example, the actin cytoskeleton can mediate the formation of the supramolecular activation cluster (SMAG) or cap (15), which in the case of leukocytes, is the cellular immune synapse (IS) (19) that exerts regulatory effects on cellular signaling (15). In particular, actin plays an essential role in the developmental maturation of T cells. Studies have shown that Pak2-mediated actin cytoskeleton remodeling is important for T cell maturation in the thymus (20). In B cells, actin is involved in the regulation of B cell receptor (BCR) clustering, IS formation, antigen internalization and presentation (21). Concurrently, BCR-mediated antigen transport in B cells is also actin-dependent (22). In addition, differences in actin kinetics in various B cell subsets contribute to their specific regulation of activation (16). In NK cells, actin can be induced to reorganize by upstream signaling molecules, triggering downstream biological processes, such as granule polarization, synapse formation and target cell lysis (23), and the density of the actin cytoskeleton mediates the cytotoxic effects of NK cells (24). Thus, the regulation of the actin cytoskeleton is essential to the function and stability of the human immune system.

**Figure 2:**
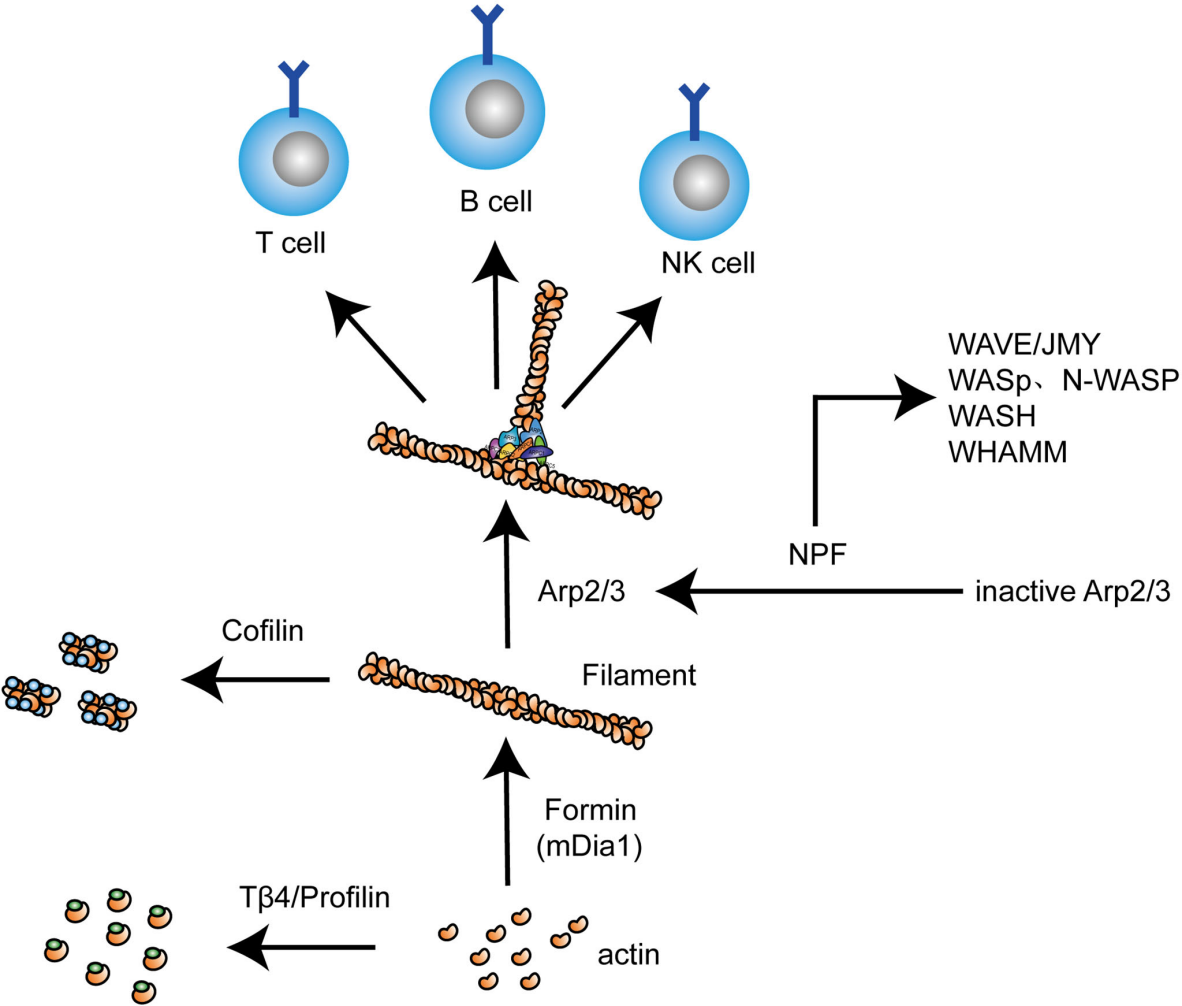
Different actin regulators play distinct roles in actin dynamics in lymphocytes. As actin-monomer-binding proteins, profilin and thymosin beta 4 (Tβ4) bind G-actin and prevent it from polymerizing, while formins act in an opposite manner, promoting actin filament polymerization. The activation of Arp2/3 complex requires NPFs including WAVE, JMY, WASp/N-WASp, WASH, and WAHMM, which is needed for branching F-actin. The severing of F-actin is mediated by cofilin. In the above process, actin undergoes dynamic changes in lymphocytes, thereby regulating cellular activity and function.

In this review, we will provide an overview of the structures and functions of several important actin regulators and how they play an important role in lymphocytes and regulating the human immune system (**Figure 2**). And we will also introduce some diseases related to the deficiency or dysfunction of these proteins and potential targets for treatment.

## 2 Structure and Basic Function

### 2.1 Actin-Monomer-Binding Proteins

#### 2.1.1 Profilin

Found by Carlsson in 1976, profilin is a 12-15 kDa protein ubiquitously expressed in eukaryotic cells of a large variety of organisms and highly expressed under hyperoxia condition (25, 26). In different organisms, profilins have a highly conserved structure: 7 β-pleated-sheets and 4 helices (27), and have 4 isoforms containing 100-130 amino acids. Profilin I is expressed in many cell types while other isoforms have tissue specificity: profilin II is brain-specific and the expression of mouse profilin III and IV is in testis, while profilin III can also be found in rat kidney (28–31). At the cellular level, profilins localize in the ruffles of peripheral lamellae and the nascent stress fibers of spreading cells, but not in peripheral belts of stationary cells in epithelioid sheets, and it is likely that profilin levels are higher in areas of active actin dynamics (32, 33). The major ligands of profilin are actin monomers, N-WASp/WAVE, PI(4,5)-P2 and poly-L-proline (34). Phosphorylation at the residue threonine 89 of profilin by protein kinase A (PKA) enables enhanced binding to actin and poly-L-proline but has no effect on phosphatidylinositol-4,5-bisphosphate (PIP2) (35, 36). Besides PKA, profilin can also be phosphorylated by Rho-associated kinase (ROCK) and dephosphorylated at Ser-137 by Protein phosphatase 1 (PP1) (37).

Profilins play an important role in cell development, motility, membrane trafficking and signal transduction *via* binding to different ligands (38). Profilin’s main function is promoting actin polymerization (39). By binding to and changing the affinity of actin monomers, profilins slow down the elongation, thus optimizing the process of actin polymerization (40). This mechanism of action speeds up nucleotide exchange, which acts opposite of thymosin-β4 (41). In addition, profilins can also enhance microtubule growth directly and take part in single cell wound repair (42, 43). Different isoforms have their specific functions. Profilin I and II play opposite roles in cell motility, migration and membrane protrusion, whereby profilin I principally enhances these processes while profilin II suppresses them (44).

As profilin can regulate actin polymerization, it plays a crucial role in cell migration (45, 46). As for its interaction with WASp-interacting protein (WIP), proteins like Mena and vasodilator-stimulated phosphoprotein (VASP) can directly bind profilin through a proline-rich Actin Based Motility (ABM) motif, such as APPPPP (47–49). WASP-WIP’s function of regulating actin polymerization may be heightened by recruiting profilin to ABM-2 motifs on WIP (45). Also, in mammalian cell lines, profilin directly binds to actin monomers to sequester them when actin barbed ends are capped, this activity assures that the G-actin pool will not be monopolized by Arp2/3 and thereby formins have the chance to enter the G-actin pool and bind actin monomers, therefore profilins hinder actin assembly mediated by Arp2/3 to promote formin activity (50, 51). Thus, profilin selectively regulates actin monomers to flow from Arp2/3 to formins and Ena/VASP (51).

#### 2.1.2 Thymosin-β4

Thymosin beta 4 (Tβ4) is a 43-amino-acid protein found to be widely expressed in thymocytes and hematopoietic cell lines, as well as in a variety of organs, such as brain, thymus, spleen, and lung (52–55). As one of the most abundant proteins in the highly conserved beta-thymosin family, Tβ4 is an important actin binding protein that binds to both the barbed and pointed ends of G-actin, which causes conformational changes in Tβ4 to spatially block actin polymerization (56, 57). This binding of Tβ4 to actin is influenced by nucleotides. It was demonstrated that the affinity of Tβ4 for ATP-actin is about 50-fold higher than that of ADP-actin (58). In addition to its role in the cytoplasm, Tβ4 also translocates to the nucleus and binds actin monomers to regulate actin dynamics (59). Overall, Tβ4’s effect on G-actin leads to an increased ratio of G-actin to F-actin in the cell, thereby regulating the cytoskeleton and affecting the biological activity of cells and tissues.

### 2.2 Wiskott-Aldrich Syndrome (WAS) Family of Proteins

Wiskott-Aldrich syndrome (WAS) is an X-linked primary immunodeficiency disease characterized by thrombocytopenia, eczema, episodes of fever, bloody diarrhea, recurrent bacterial infections, innate and adaptive immune deficiency, and a high rate of autoimmunity and malignancies (60, 61). The disease is due to mutations of the gene which encodes the WAS protein (WASp) (62). WASp was the first identified member of a family of proteins comprised of WASp/N-WASP, SCAR/WAVE, WHAMM/JMY/WHAMY, and WASH subfamilies (63). WAS proteins are nucleation-promoting factors (NPFs) that activate Arp2/3 to nucleate branched actin filaments in response to extracellular signals. This plays an important role in many cellular processes that happen at the cell surface, such as cellular motility, endocytosis, exocytosis and intracellular signal transduction (64). WAS family proteins are also present in the nucleus, where they regulate transcription and remodel chromatin (65). Distinct WAS family proteins participate in different cellular processes, therefore, they differ from each other in the structure of their N-terminal portions. However, contrary to their diverse N termini, all WAS family proteins possess an identical C-terminal structure, the verprolin homology (WH2)/connecting peptide/acidic domains (VCA) domain, which mediates interaction with the Arp2/3 complex and actin (**Figure 1**) (64). It was also reported that WAS family proteins function as polymerases, accelerating elongation of uncapped actin filaments (66). In summary, WAS family proteins have the following functions: nucleating branched actin filaments, linking actin networks to membranes, mediating transcription and chromatin remodeling, and accelerating filament elongation.

#### 2.2.1 Wiskott-Aldrich Syndrome Protein (WASp)

WASp is a 502-amino-acid protein expressed exclusively in hematopoietic cells (62), which has several domains with different functions. The pleckstrin homology (PH) domain is close to the N-terminal region and is involved in the localization of WASp through interactions with other proteins or lipids, such as PIP2. The PH domain is very important since missense mutations in this region lead to severe diseases (67). The PH domain overlaps the Ena/VASP homology 1 (EVH1) domain (also known as WH1 domain), which constitutively interacts with the proline-rich region of the WIP in resting cells (68). The EVH1/WH1 domain is followed by a basic region (BR), a GTPase-binding domain (GBD), a poly-proline region (PP) and a VCA domain in the C-terminal (**Figure 1**) (60). In the inactive state, WASp is in an auto-inhibited conformation in which the GBD of WASp conceals the VCA domain. This prevents the interaction between WASp and Arp2/3 and inhibits actin polymerization. However, upon receptor activation mediated by extracellular stimulation, such as T cell receptor (TCR) and BCR signaling, WASp is recruited to the signaling site at the membrane and transformed to the active conformation (69). This process is induced by activated GTP-bound cell division control protein 42 homolog (Cdc42), which binds to the GBD and transforms WASp to an open-activated structure, allowing it to interact with Arp2/3 and promote actin polymerization. Additionally, proteins with Src homology 3 (SH3) domains, including Grb2, p47nck, Fyn, and Lck, combine with Cdc42 to enhance WASp conformational changes. Furthermore, Src-family kinases, such as Lyn and the Tec family of cytoplasmic tyrosine kinases, including Btk, Tec, and Itk, phosphorylate WASp at Tyr 291 (Y291) in a Cdc42-dependent fashion, which modulates the affinity of WASp for Cdc42 and later targets WASp for degradation *via* a ubiquitin-dependent proteasomal pathway (60, 68, 70). After activation of WASp, actin branching is mediated by the Arp2/3 complex, which is initiated by several steps. First, the V/WH2 segment of VCA binds to G-actin monomers, while the CA segment binds two sites on the Arp2/3 complex. These WASp-recruited actin monomers will be the first actin subunits added onto the newly branching filament. Second, the WASp-Arp2/3 complex undergoes a conformational change where two actin-related subunits in the complex, Arp2 and Arp3, are transformed into a short pitch conformation, which imitates an actin dimer within a filament. Finally, WASp interacts with an existing actin filament to initiate nucleation, which ensures only branched actin filaments are generated (64). After the filament branching process is finished, activated WASp is down-regulated by ubiquitination and proteasome degradation. In the resting state, WIP can inhibit WASp degradation by concealing its ubiquitination sites (69). Following activation, WIP undergoes phosphorylation and conformational changes, which exposes WASp’s ubiquitination sites in the WH1 domain. After ubiquitinated by E3 ligases, c‐Cbl and Cbl‐b, WASp is targeted for protease calpain degradation (68).

#### 2.2.2 Neuronal Wiskott-Aldrich Syndrome Protein (N-WASP)

Another WASP family protein that plays a crucial role in cells is the Neuronal Wiskott-Aldrich Syndrome Protein (N-WASP). Unlike WASp, which is expressed exclusively in hematopoietic cells, the mRNA of N-WASP has been found to be widely localized in several organs including brain and colon (71). N-WASP has 50% homology with WASp at the protein level (71, 72), with the main structural difference being that N-WASP contains two verprolin homology sequences in the VCA structural domain (VVCA), which allows it to bind to Arp2/3 more efficiently than WASp (73). The structural similarity of N-WASP with WASp allows for their similar regulatory roles in cells. For example, expression of the N-WASP gene in WASp-deficient hematopoietic stem cells can partially rescue the signaling defect in T cells (74). However, mutations in the WASp gene predisposes someone to WAS syndrome, while N-WASP deletion is embryonic lethal (75). This suggests that the two proteins do not function in exactly the same way, which is supported by the experimental finding that N-WASP lacking the WASp-specific I30 region cannot rescue the chemotactic defects of WASP knockout Jurkat T-cells (76).

The activity of N-WASP is synergistically regulated by Cdc42 and PIP2. In the inactivated state, the VCA domain of N-WASP binds to the GBD and is in a self-inhibited state that cannot interact with Arp2/3 (77). N-WASP has binding sites for Cdc42 and PIP2. Binding of one of these molecules induces a conformational change in N-WASP that exposes the binding site for the other, which allows for both molecules to bind and activate N-WASP (78). N-WASP can also be activated by Src family kinases through phosphorylation (79) and proteins containing SH3 structural domains (such as cortactin) are also able to bind to N-WASP and activate its Arp2/3 binding capacity. Phosphorylation of cortactin by Erk facilitates this process, while phosphorylation by Src inhibits it (80). N-WASP and Cdc42 play a unique role in the formation of cellular filopodia (81) and the complex formed by N-WASP with WIP and Nck is necessary for the construction of dorsal ruffles (82).

#### 2.2.3 Wiskott-Aldrich Syndrome Protein (WASp) and SCAR Homologue (WASH)

WASH is one of the novel members of the WASP protein family and has been found to be distributed in several human tissues including blood cells (83). The C-terminal structural domain (VCA domain) in WASH proteins, similar to WASP, enables it to function as a downstream effector molecule of Rho in Drosophila and as an Arp2/3 activator (84). Additionally, its unique N-terminal structural domains, including WASH homology domain 1 (WAHD1) and tubulin-binding region (TBR), give it the ability to interact directly with tubulin (85). Such structural properties allow WASH to localize to multiple types of endosomes and help maintain the stability of endosome morphology and the proper recycling pathway, especially for membrane surface receptors (86, 87). Also, WASH plays an important role in processes such as autophagy and cell differentiation (88, 89).

### 2.3 Nucleation Proteins

#### 2.3.1 Arp2/3 Complex

The Arp2/3 complex is an important actin filament nucleation factor that was first purified from Acanthamoeba (90). It is evolutionarily conserved in most eukaryotic cells and consists of seven subunits (91), two of which, Arp2 and Arp3, are actin-related proteins that are structurally similar to actin (92). The other five subunits are ARPC1 through ARPC5, of which ARPC2 and ARPC4 form the center of the complex (93) called the clamp subunit. Recent studies have confirmed that the composition of the Arp2/3 complex was not constant (94), for instance, in humans, ARPC1 contained two isoforms, ARPC1A and ARPC1B, and different Arp2/3 complexes could play different biological roles.

The most basic function of the Arp2/3 complex is nucleation and branching of the actin filament. Previous *in vitro* studies have shown that the Arp2/3 complex itself had a weak nucleation capacity (95) and required a class of proteins called nucleation-promoting factors (NPFs) for activation. NPFs can be divided into two types. Type I NPFs, including WAVE, WASp, N-WASP, WASH and junction-mediating and regulatory protein (JMY), contain a VCA structural domain (i.e., the verprolin-homology domain, the cofilin-homology domain and the acidic domain) that interacts with the Arp2/3 complex and binds to actin monomers. Type II NPFs, such as Cortactin, contain an acidic region that binds to Arp2/3 and weakly activates the Arp2/3 complex, as well as having effects such as stabilizing the filament branching structure (96). It was confirmed that the Arp2/3 complex contained two different activation sites for NPFs and functioned differently during activation (97), thus creating a prerequisite for the synergistic activation of the Arp2/3 complex by cortactin and WASP family proteins (98). During the activation of the Arp2/3 complex by NPFs, the complex binds to existing actin filaments (mother) and undergoes intra-subunit and inter-subunit changes (99). The one conformation is induced by clamp twisting within the subunit, which transforms the original twisted conformation of Arp into a flat conformation. The other conformation is created when the Arps form a short-pitch conformation, which mimics the end of the actin filament. As a result, the Arp2/3 complex initiates the formation of new filaments (daughter) on the mother chain and facilitates the formation of the actin branching network. Besides the activation of the Arp2/3 complex by proteins like NPFs, there are a variety of intracellular inhibitors that negatively regulate it. For instance, the binding of cofilin, an actin filament severing protein (discussed below), to the actin filament reduces the affinity of Arp2/3 for F-actin, thereby contributing to the detachment of Arp2/3 from the filament and debranching (100). This reduction in affinity may be achieved through direct competition of cofilin or conformational changes of the filament. Additionally, Glia Maturation Factor (GMF), which belongs to the actin depolymerizing factor homology (ADF-H) family with cofilin (101), was found to bind to Arp2/3, inhibiting its nucleation and inducing debranching (102, 103). Ydenberg et al. subsequently proposed that GMF mediated debranching *via* its two binding sites by binding the first actin subunit on the Arp2/3 and the filament branch (104). The process involved is similar to the severing mechanism of cofilin (104, 105), with GMF in coordination with cofilin regulates the debranching and severing of filaments. Coronins (discussed below) are also known to have an inhibitory effect on the branching of Arp2/3. Studies performed in different subgroups of coronins have confirmed their function in binding to Arp2/3 and inhibiting its actin nucleation activity (106–108), and they have synergistic effects with GMF in this regard (109). Similarly, the acidic motif of the newly discovered protein Arpin identified by Dang et al. can competitively inhibit the VCA domain of Arp2/3, thereby inhibiting the activity of the complex (110). Studies on the structure of Arp2/3 further confirmed that binding to Coronin, GMF and Arpin led to a shift of Arp2/3 into an open/inactive conformation, thus providing an explanation for their inhibitory effect (109). Moreover, the binding and hydrolysis of nucleotides affects the activity of the Arp2/3 complex (111, 112), and it was found that the hydrolysis of ATP played an important role in debranching (113). Furthermore, the effect of phosphorylation regulation on the Arp2/3 complex activity is of important interest (114–116), thus the regulation of Arp2/3 is a mutually coordinated and sophisticated process, which creates the prerequisite for its regulation of the actin cytoskeleton.

The nucleation effect of the Arp2/3 complex on actin makes it an important regulator of the cytoskeleton. Thus, the Arp2/3 complex plays a significant role in a variety of cellular activities. Experiments on *Dictyostelium* (117) and mice (118) suggest that defects in the Arp2/3 complex may be lethal. For cell migration, the Arp2/3 complex is localized in cell protrusions, including lamellipodia and pseudopodia (119, 120), which are critical for cell motility. Moreover, the Arp2/3 complex has been found to be essential for cell adhesion (121) and it also plays a key role in cellular endocytosis (122, 123), whereby disruption of Arp2/3 complex activators can lead to defects in endocytosis (124). In addition, the Arp2/3 complex is involved in Golgi-associated membrane transport processes (125, 126) as well as cellular phagocytosis (127), which is of great significance in the immune response. And recent studies have revealed that the WASH and Arp2/3 complexes were associated with centrosomes and that the actin nucleation mediated by them might function in cell mitosis (128, 129).

### 2.4 Actin Filament Polymerases

#### 2.4.1 Formins

Formin homology proteins (Formins) are a family of highly conserved proteins that are widely found in eukaryotes and are involved in the regulation of the cytoskeleton (130). Members of the Formins family are morphologically diverse, with 15 different formin proteins present in humans (131), but relatively few formin proteins in yeast. Nevertheless, there are regions of similarity between most of these proteins, including formin homology 1 (FH1) and formin homology 2 (FH2) domains, which are key regions determining the regulation of actin polymerization by formin proteins. The FH1 domain is located in the N-terminal to the FH2 domain and contains multiple repeating units of polyproline that can act as a profilin ligand (132, 133). Polyproline segments are of variable length with non-proline residues inserted in between them, which are also important in the role of the FH1 structural domain (132, 134).

**Figure 3:**
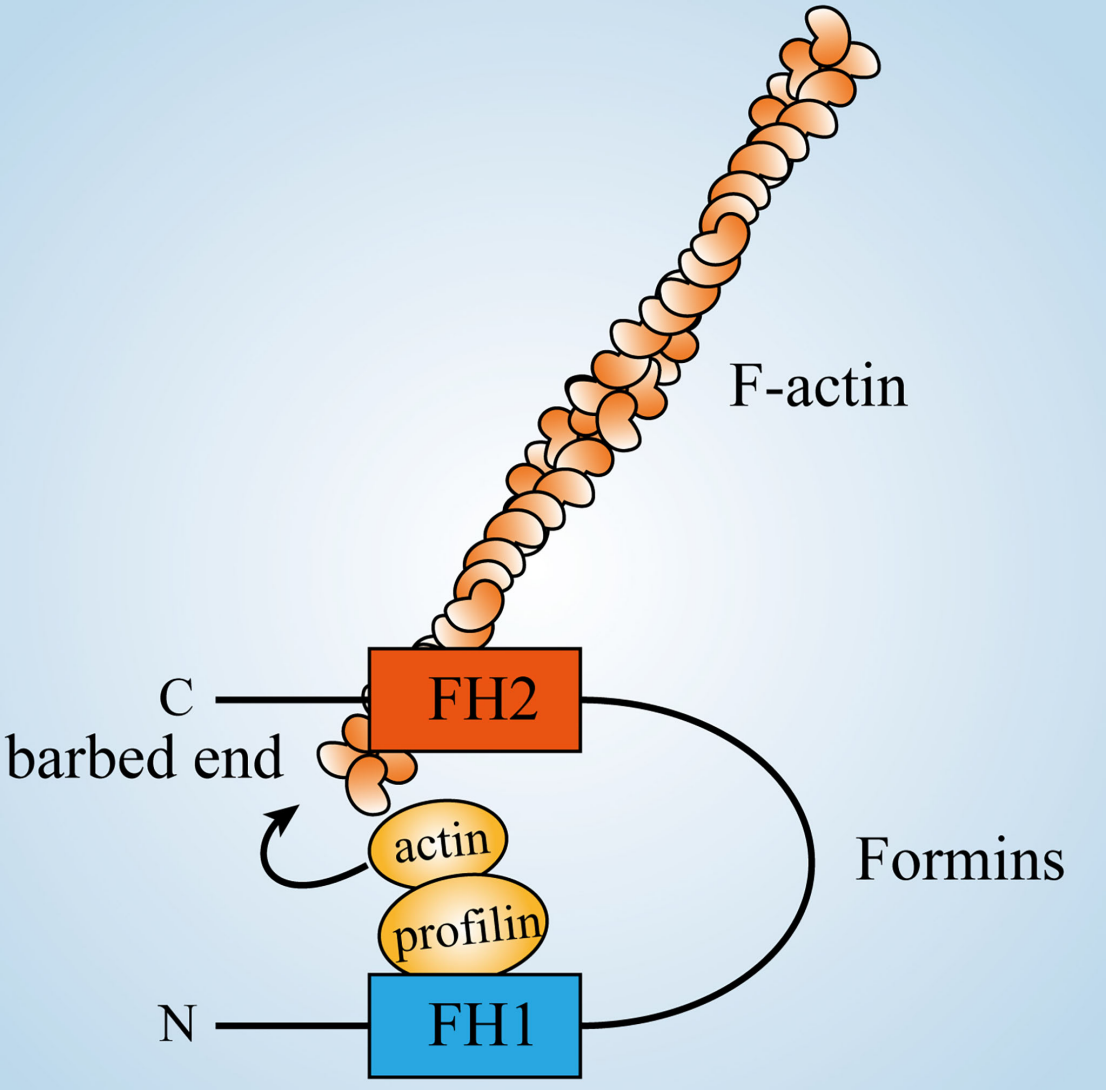
Formins’ role in regulating actin dynamics. The FH2 domain of formins close to the C-terminus can bind to the barbed end of filaments, while the FH1 domain near the N-terminus can bind to profilin-actin, thus increasing the concentration of actin monomers around filaments and positively promoting their polymerization.

Profilin is an actin monomer-binding protein and its binding to the FH1 domain increases the profilin-actin affinity to the FH2-associated barbed end and helps in the rapid elongation of microfilaments (**Figure 3**) (135, 136). The crystal structure of the FH2 domain is in the form of a tethered dimer (137, 138), which is consistent with its ability to bind actin nuclei or filament barbed ends (139). The FH2 domain assists in nucleation by stabilizing actin oligomers (140) and can mediate the addition of G-actin to the barbed end of a filament while preventing the binding of capping proteins (141). In addition to the above two structural domains, some formin protein sequences contain some or all of the GBD, Diaphanous Inhibitory Domain (DID) and Diaphanous Autoregulatory Domain (DAD). The role of Formins in regulating the formation of unbranched actin filaments affects activities such as formation of cellular protrusions (7, 142, 143) and cytokinesis (144). In addition, Formins can regulate microtubule dynamics and link them to actin dynamics to coordinate cytoskeletal activities (145–147).

### 2.5 Severing Proteins

#### 2.5.1 Cofilin

Cofilin, a highly conserved protein with a molecular weight of 19 kDa, was first identified in chick brains and can bind to actin monomers on filaments in a 1:1 molar ratio (148, 149). As research progressed on cofilin, it was found to belong to the actin-depolymerizing factor (ADF)/cofilin family, which is a family of actin-binding proteins mainly containing three isoforms (Cofilin 1, Cofilin 2 and ADF). The structure of cofilin is relatively conserved consisting mainly of an ADF-H domain (UniProtP23528 and UniProt Q9Y281) which was found to interact with G-actin, filamentous actin or the Arp2/3 complex (150). And the tridimensional structure has two pairs of α-helices at the periphery, with a hybrid β-sheet sandwiched in between (151). It is currently believed that all eukaryotic cells contain at least one ADF/cofilin protein (152). In mammals, two cofilin isoforms are known. One is the NM-type (CFL1), which is widespread in non-muscle tissues, and the other is the M-type (CFL2), which is mainly expressed in muscle cells, but can also be seen in other tissues like testis (153, 154). For humans, the genes encoding CFL1 and CFL2 are localized on chromosomes 11 and 14, respectively (155).

As a member of the actin-binding protein family, the primary role of cofilin is to modulate actin dynamics. The cofilin protein contains a G/F site at the C-terminus and an F site at the N-terminal end (156), giving it the ability to bind to actin monomers (G-actin) or F-actin, in which it severs and depolymerizes actin filaments. Cofilin was initially thought to bind to some of the acidic residues at the N-terminal of actin by electrostatic interaction (157). Subsequent studies supported this by finding that cofilin binds to F-actin along the two initial helices between neighboring subunits (158), and that the ADF-H domain of cofilin binds between actin subdomains 1 and 3 to insert into its hydrophobic cleft (159). *In vivo* studies have shown that low concentrations of cofilin prefer to function in severing F-actin, while high concentrations tend to facilitate actin nucleation (160). A similar study also found that filaments are stabilized in the saturated cofilin state (160). However, this rule does not always hold. For example, in the thymus, it has been found that during cofilin saturation, cofilin was still able to depolymerize filaments in the presence of actin interacting protein 1 (Aip1), another important actin regulator (161). Many factors regulate the activity of cofilin. Phosphorylation or dephosphorylation of cofilin is a critical step in determining its activity (162), and phosphorylation at Ser-3 inactivates cofilin (163). pH level also affects the polymerization or depolymerization activity of cofilin on actin (164). In humans, depolymerization of actin mediated by cofilin increased when the pH was elevated from 6.5 to 8.0 (165). A similar study also found that cofilin favors binding to F-actin in a neutral or weakly acidic environment, while the extent of binding is greatly reduced in a weakly basic condition (166). Moreover, cofilin activity can also be influenced by phosphoinositide, where PIP2 is capable of inhibiting cofilin from binding to actin (167) and nucleotides, which cofilin has a higher affinity for ADP-actin compared to ATP-actin (168). It has also been proposed that the pH regulation of cofilin might be achieved through binding to phosphatidylinositol (169).

#### 2.5.2 Coronin

Coronin is a 55 kDa protein that was first purified from growth-phase *Dictyostelium Discoideum* cells by E.L.deHostos and his co-workers. They named it “coronin” because of its interaction with the crown-shaped projections on the dorsal cortex of the cell. Through preliminary co-sedimentation experiments, they found coronin could colocalize with actin filaments (170). Different types and subtypes of coronins have been discovered and classified, however in this review we use the widely accepted classification method to divide them into 3 types: type I includes coronin 1A, coronin 1B, coronin 1C and the newly-found coronin 6. Type II includes coronin 2A and coronin 2B, and type III is coronin 7 in humans, or POD in nematode and *Drosophila melanogaster* (106). Up to now, coronin research has mainly focused on coronin 1A and less on the other types. Coronins are highly conserved proteins and the basic structure of the coronin family contains a 7-bladed β-propeller formed by 5 WD repeats on the N-terminal, a classical heptad coiled-coil domain on the C-terminal and an irregular secondary domain in the middle (171, 172). The N-terminal has phosphorylation sites, which mediate protein interactions and the coiled-coil domain regulates homo-oligomerization to help coronin 1A form a tripolymer structure (173, 174). Unlike other coronins, coronin 7 has two classical WD domains, which means two β propellers, but it has only one coiled-coil domain with an additional acidic domain, whose structure is similar to the acidic domain in the SCAR/WASP (172).

Encoded by the CORO gene, researches have shown that coronins are expressed in all eukaryotes, but as for the tissue specificity, coronin 1A is mainly expressed in hematopoietic tissues and immune cells (175). Coronin 1B and coronin 1C are ubiquitously expressed in different tissues and may be involved with cell migration (106). Type II coronins can be found only in vertebrates, they have different C-terminal structure from type I. Coronin 2A is expressed in testis, ovary, uterus and brain, while coronin 2B expression is mainly in the brain (176). Coronin 7 is found in mammals and POD in Caenorhabditis elegans, however being of similar genes, both localize and function within the Golgi complex in all kinds of cells (176).

Almost all the coronins belong to the actin filament-crosslinking and bundling protein family and they concentrate in the actin-rich areas on the cell membrane (177). The fundamental function of coronins is facilitating the actin depolymerization *via* interacting with Arp2/3 and actin filaments, and the binding site to actin is the KXRHXX-motif located near the N-terminal and the WD domain (178). Coronins, cofilins and WD repeat-containing protein 1 (WDR1) form an organized unit for the regulation of actin. The binding of coronin with actin enhances the binding of cofilin to actin as the filament twist is changed. Additionally, the cooperation between coronin and slingshot-1L increases the activity of cofilin (106, 179). In yeast, coronins bind Arp2/3 directly by a coiled-coil domain and suppress the nucleating activity of Arp2/3 (107). This mechanism is properly regulated by phosphorylation of protein kinase C (PKC) (173). However, recent studies demonstrated that while a high concentration of coronin restrains Arp2/3, a lower concentration of coronin could promote Arp2/3 nucleation (180).

#### 2.5.3 WD Repeat-Containing Protein 1 (WDR1)

Aip1, also known as WDR1, is a conserved protein that belongs to the WD repeat domain-containing proteins, whose protein structure is mainly composed of two connected seven-bladed β-propellers (181). WDR1 alone has little effect on actin dynamics, but it assists cofilin in severing/depolymerizing of actin filaments (182, 183), a process that often also involves a third protein, coronin (discussed it above), which acts synergistically to promote the severing of filaments (184, 185). At the same time, studies in leukocytes have found that the actin-promoting effect of WDR1 is also influenced by caspase-11, the deletion of which leads to impaired motility of immune cells, including T cells (186).

## 3 Functions in Lymphocytes

### 3.1 T Lymphocytes

T cells are derived from the thymus. Mature T cells settle in thymus-dependent areas of peripheral immune organs, where they mediate adaptive cellular immune responses and also play an important secondary role in thymus-dependent antigen-induced humoral immune responses. The activity of actin regulators in T cells affects the development, motility, and functional effects of T cells.

#### 3.1.1 Development

Bone marrow T cell progenitors locate to the thymus, where they develop into mature T cells *via* stages of differentiation, they then enter the peripheral lymphoid organs by the blood circulation. At this time, the naive mature T cells that come in contact with antigen will proliferate and differentiate into effector T cells, regulatory T cells or memory T cells that all have different functions (187). This is a very complex process in which various actin regulators are involved.

Earlier studies found that Tβ4 induces phenotypic changes in the human T cell line Molt-4 and may be involved in early cell differentiation (188). As an important member of NPFs, WASp also has an important regulatory role on T cell development. It has been shown that high expression of WASp inhibits the growth of T-cell lymphoma, while the loss of WASp inhibits T cell activation processes including decreasing T cell proliferation induced by TCR stimulation and preventing cytokine polarization and secretion (189). Specifically, CD8^+^ T cells from WASp-deficient mice are hyperactive with increased cytokine production, however these cells are also insufficient in CD8^+^ memory T cells differentiation and have increased apoptosis through upregulation of the Fas pathway. This further implicates a role for WASp in the survival and differentiation of CD8^+^ T cells (190). Additionally, double knockout (DKO) mice compared to single knockout of WASp or N-WASP mice, exhibit more pronounced abnormal thymic development and impaired T cell development, which is due to defects in cytoskeletal reorganization and migration (191). This indicates that N-WASP exhibits a synergistic effect with WASp during T cell development. WASH is required for the efficient proliferation of T cells after responding to CD3/CD28 signaling, which is related to its function in regulating the intracellular transport of T cell surface molecules, including TCR (192). Concurrently, the lack of coronin 1A leads to decreased naïve T cells and developments of severe combined immunodeficiency (SCID) (193). This is because coronin participates in the activation of calcium-calcineurin signaling that maintains the survival of naïve T cells (194), which was found have a similar effect on peripheral T cells (195). Also, abrupt reduction in the number of peripheral αβT cells and impaired late development of thymocytes caused by coronin1A defects indicate its significance in terminal T cell differentiation (196). For WDR1, it has been previously shown that defective expression of the WDR1 gene predisposes someone to autoinflammatory disease and thrombocytopenia, which are mainly manifested by abnormal neutrophil behavior (197, 198). A subsequent study reported a reduced rate of follicular helper T (Tfh) cells in patients with defective WDR1 gene expression and also showed a decreased Ca^2+^ response in TCR proximal signaling, suggesting that WDR1 may have an effect on T cell development and TCR signaling (199).

#### 3.1.2 Cell Migration

Migration is one of the bases for accurate targeting of T lymphocytes to sites of infection (200). In response to chemokine stimulation, T cells can polarize, extend lamellipodium at the leading edge and produce a uropod at the rear end. Integrin alpha 4 beta 1 and alpha L beta 2 induce directional with T cell motility (201), which several actin regulators play a role in.

The migratory capacity of T cells partly depends on the production and location of actin and cofilin, which can be illustrated by the experimental fact that cofilin mRNA is distributed at the leading edge of migrating cells (202). Meanwhile, studies in Jurkat T cells revealed that cofilin is phosphorylated by the spatiotemporal regulation of LIM domain kinase (LIMK) and SSH1L, and this physiological process is crucial for SDF-1α-induced T cell migration (203). One possible regulatory mechanism is that following G-protein-coupled receptor stimulation, LIM-Kinase1 activity is inhibited in T cells *via* the Ras-MEK pathway to promote the dephosphorylation of cofilin (204). Activated cofilin may function at the leading edge of the cell to sever the F-actin, increasing the number of barbed ends to promote nucleation of actin polymerization there (152, 205). When the activity of MEK or cofilin is inhibited, lamellipodia extension is impaired, inhibiting the cell migration rate (206). This mechanism may play an important role in the localization of T cells during the immune response (204).

Another study on Jurkat T cells found that cell spreading on anti-CD3 sheet-like structures was inhibited by lack of Arp2 (207). Furthermore, preventing actin polymerization mediated by WASp/Arp2/3 inhibits T cell chemotaxis (208). This is due to the Arp2/3 complex being an important regulator of membrane protrusion formation, including lamellipodia (209), which is the main mode of T cell motility. Studies have found that in Arp3-impaired CTL, the cell motility pattern changed from lamellipodia-based to blebbing-like migration, a mode of movement resulting from the detachment of the plasma membrane from the actomyosin cortex (210), which decreases the migration speed (211). The different motility characteristics exhibited by T cells under different antigen affinities depends on the regulation of the Arp2/3 complex (212), which allows T cells to decelerate when encountering suitable antigens and is important to T cell bioactivity.

**Figure 4:**
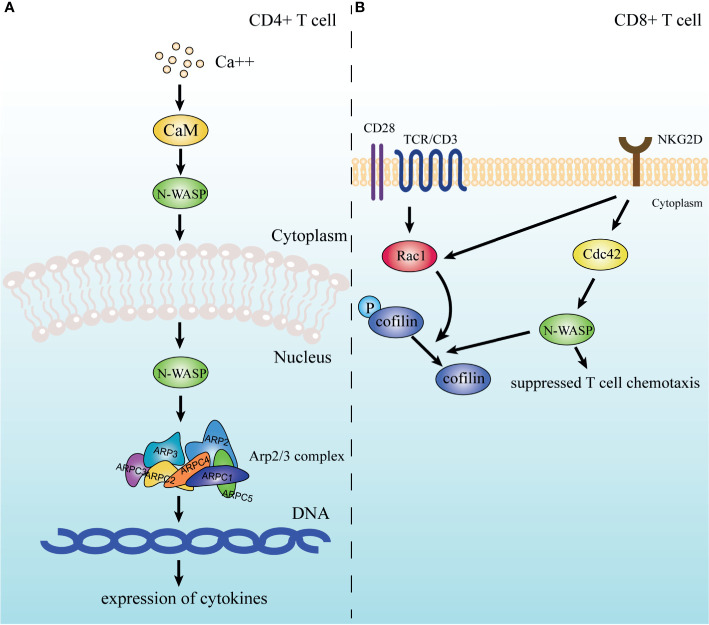
N-WASP is involved in signaling pathways of T cells. **(A)** In CD4^+^ T cells, N-WASP is activated by CaM and enters the nucleus to coordinate the Arp2/3 complex to participate in the expression of cytokines. **(B)** In CD8^+^ T cells, N-WASP receives signals from NKG2D and participates in downstream cofilin dephosphorylation while also mediating the inhibition of cellular chemotaxis.

Unsurprisingly, N-WASP, the regulatory protein of the Arp2/3 complex, is also involved in T cell motility. An earlier study found that Cdc42 interacting protein 4 (CIP4), a protein that binds to Cdc42 and WASP/N-WASP and is involved in cytoskeletal regulation, is necessary for integrin-dependent T-cell migration, implying the participation of N-WASP in T cell motility (213). Specifically, in CD8^+^ T cells, N-WASP is a downstream effector molecule of NKG2D, which is a transmembrane receptor expressed mainly on CD8^+^ T cells and NK cells. And N-WASP is involved in the inhibitory effect of NKG2D on T cell chemotaxis under the regulation of activated Cdc42 upon CD3/NKG2D activation, and this pathway may also involve the downstream dephosphorylation of cofilin (**Figure 4**) (214).

Formin-like-1 (FMNL1) and mDia1 are known to be the predominantly expressed formin proteins in T cells (215). FMNL1-mediated cytoskeletal dynamics allows T cells to undergo shape changes for adapting to the environment, helping them to complete transendothelial migration (TEM) and translocation to sites of inflammation to exert immune effects (216). RhoA-regulated mDia is expressed in activated T cells and affects the functional exertion of Rac1 through the regulation of actin equilibrium, however overexpressed mDia negatively regulates the motility of T cells (46). Additionally, by studying T cells from p140mDia1-encoding knockout mice, Eisenmann et al. found that this protein is necessary for proper T cell development and chemotactic movement (217). Similarly, mDia1 deficiency inhibits T cell proliferation and chemotaxis induced migration, thus impairing the relocation of T cells to secondary lymphoid organs (218). Further studies revealed that mDia1 regulates microtubule dynamics and helps in T cell adhesion and translocation by inactivating glycogen synthase kinase (GSK) 3β and protecting adenomatous polyposis coli from phosphorylation (219).

The regulatory effects of Coronin and WDR1 on actin allow them to also influence T cell motility. Coronin1A helps T cell egress from the thymus and the coronin knock-out phenotype in mice shows deficiency in T cell migration, whereby there is abnormal accumulation of F-actin and failure of lamellipodia formation (106, 220). Furthermore, the enhanced effect of WDR1 on cofilin activity was shown to contribute to the chemotaxis of Jurkat cells, and also WDR1 is essential for their normal morphological maintenance and changes, which can be achieved by promoting the remodeling of the actin cytoskeleton (221).

#### 3.1.3 Immune Synapse

T cells become activated when their T cell receptors (TCRs) bind specifically to antigen presented by major histocompatibility molecules (MHCs) on antigen-presenting cells (APCs). This binding to antigens induces signaling by the TCRs that lead to activation of signaling pathways involved in promoting T cell functions. This activation process requires signal transduction and amplification, which is supported by the stable platform created by the IS. The IS is a membrane structure formed on the surface of T cells at the contact site with APCs. The center of the IS is a cluster of TCRs with adhesion molecules surrounding the periphery (222, 223). This structure is also known as a SMAC, which includes the central supramolecular activation cluster (cSMAC), the peripheral SMAC (pSMAC) and the distal SMAC (dSMAC) (224, 225). IS formation is a dynamic process that depends on the activity of the actin cytoskeleton (223, 226), and therefore the contribution of actin regulators in this process is critical. Since IS formation is closely related to TCR signaling, which will be discussed below, we provide a brief overview of the actin regulators involved.

During IS formation, constant actin depolymerization and repolymerization contribute to the aggregation of related molecules, including TCR, LFA-1, and CD45. A number of experiments have shown that multiple actin regulators were important in IS formation. First, it was demonstrated that IS formation was halted with the inhibition of cofilin depolymerization activity (227). Second, the Arp2/3 complex was found to be important in the actin polymerization that is essential for IS formation. This was determined in patients with combined immunodeficiency (CID) that have a homozygotic mutation in the gene ARPC1B, which results in a defect in the development of the T cell IS (228–230). Additionally, ARPC3 is an essential protein involved in the TCR-related vesicular transport that enables TCR recycling between the intracellular and plasma membranes to continuously supply TCRs for activation and IS formation (231, 232). In this process, ARPC3 acts in concert with proteins such as IFT20 to regulate the assembly of a functional IS (233). Furthermore, the Arp2/3 complex is degraded intracellularly by GRAIL *via* Lys-48 and Lys-63 ubiquitination to regulate IS formation and keep T cells in a state of anergy (234), which is essential for immune tolerance of T cells. Along with Arp2/3, WASp also takes part in actin assembly and IS stabilization. It was found that downregulation of WASp causes actin foci to disappear and the symmetry of the IS to be destroyed (57). And WASp/N-WASp can function with Nck to regulate the interaction and movement between LAT and actin, which contributes to the formation of the IS (58). Similarly, Formins promote the formation of TCR microclusters through polymerizing F-actin during the early stages of IS formation in T cells (217, 235), and the aggregation of actin by Formins at the IS distal edge is needed to form the actin arc at the IS, which affects activities such as adhesion and signal transduction of T cells (236). A recent study also found that PKCδ-dependent phosphorylation of the formin protein, FMNL1β, negatively regulated F-actin polymerization at the IS and thus facilitated microtubule-organizing centers (MTOC) polarization (56). Furthermore, during IS formation in T cells, centrosome polarization requires the formation of stable and detyrosinated microtubules, which is a process mediated by the FH2 domain of INF2 (55). Thus, although the cytoskeletal components involved in IS formation is predominantly actin, other components may also be involved in this process and requires further research.

#### 3.1.4 TCR Signaling

TCR signaling is initiated after T cells recognize a specific MHC-peptide complex (pMHC) on the APC. This process consists of interaction of receptors, followed by receptor activation and intracellular signal transduction. Coreceptors on T cells (CD4 or CD8), bind to the non-peptide-like region of MHCs, inducing recruitment and phosphorylation of tyrosines in the immunoreceptor tyrosine-based activation motif (ITAM) of the cytoplasmic segment of CD3 molecules. The process of phosphorylation is mainly mediated by Lck, a protein tyrosine kinase, which is an essential molecule for transmission of the external activity of the TCR into an internal signal. Phosphorylated ITAMs provide a binding site for ZAP-70, which is activated and continues the cascade of phosphorylation of downstream molecules and initiates several signaling pathways, including the PKC pathway, small G-protein pathway, and Ca^2+^ pathway, to regulate T cell activity (237–239). The understanding of the TCR signaling pathway involves millions of studies, and here we focus more on the role of actin regulators in it.

**Figure 5:**
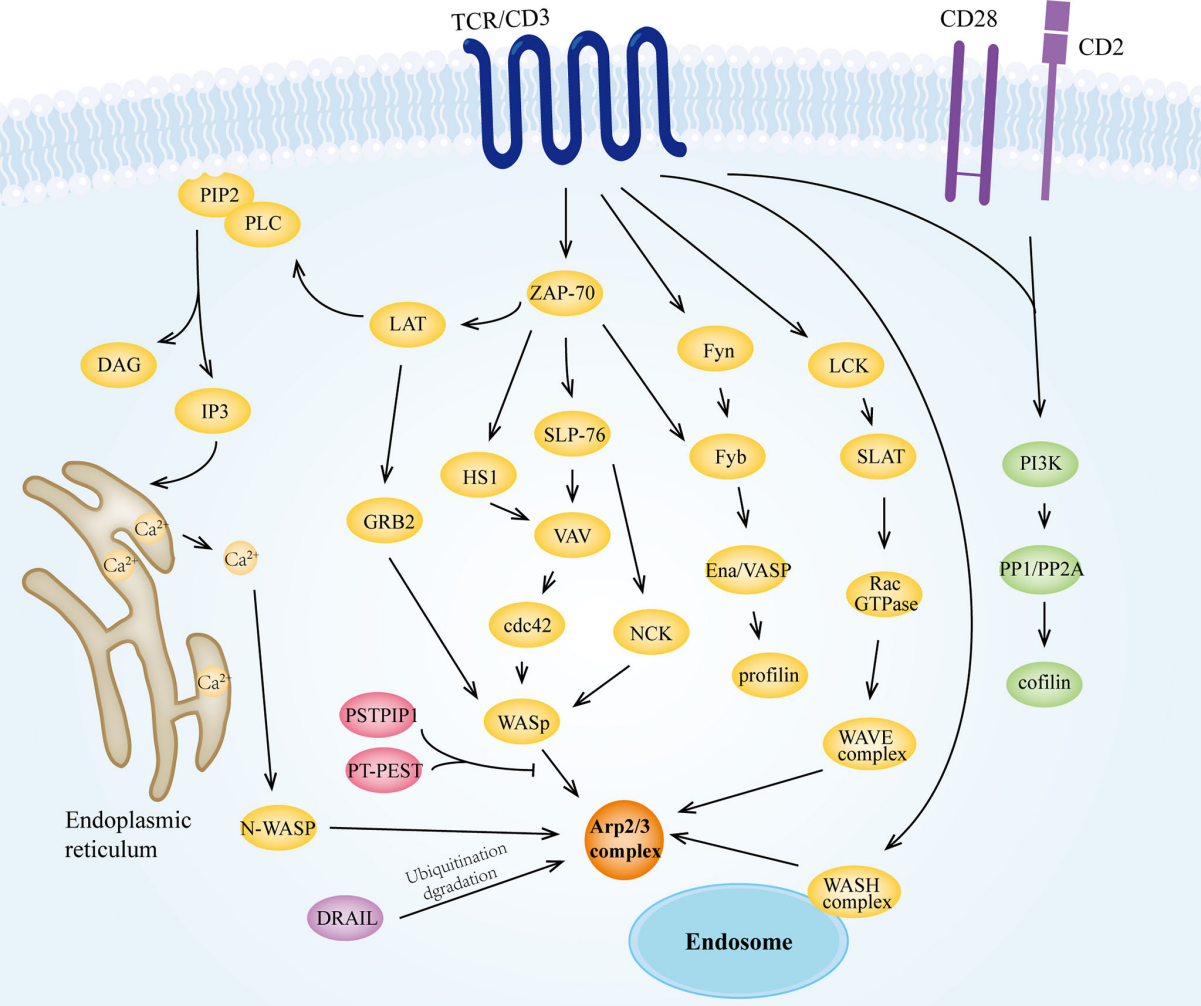
The Arp2/3 complex is involved in TCR signaling. Upon receiving signals from MHC molecules, TCR/CD3 can transmit stimuli to the Arp2/3 complex *via* tyrosine kinases such as Fyn, LCK, and ZAP-70. In this process, WASp family proteins play an important mediating role as direct activators of the Arp2/3 complex. The activated Arp2/3 complex has the ability to promote branched filament polymerization, which together with other T cell signaling-induced activation of actin regulators (e.g., cofilin and profilin) modulates the actin cytoskeleton, which is important for the biological activities of T cells, especially for IS formation.

The role of the Arp2/3 complex in TCR signaling has long been of interest. Several studies have shown that the Arp2/3 complex maintains TCR downstream signaling (233, 240). A more definitive study suggests that deletion of APCR2 in T cells leads to reduced levels of cell surface TCRs, and that the Arp2/3 complex is also involved in the endosomal transport of TCRs, thus affecting the proximal TCR signaling (241). Indeed, Arp2/3 complex-mediated actin dynamics is a critical step in multiple pathways, and more often as an effector molecule of the distal TCR signaling, linking it to alterations in the actin cytoskeleton (**Figure 5**). Studies have indicated that after recognition of pMHC by TCR/CD3, stimulation is delivered to downstream tyrosine kinases like Fyn, LCK, and ZAP-70 (242), which then phosphorylate downstream molecules like LAT, SLP-76, Fyb, SLAP-130 (243), that ultimately activate the Arp2/3 complex. Multiple activators of the Arp2/3 complex, including NPFs, play significant roles in the signaling pathway. For instance, at the IS of T cells, the GTPase-binding domain of WASp binds to Cdc42-GTP, releasing the VAC domain and promoting the function of the WASp-Arp2/3 complex (70, 244). Also, PIP2 produced by TCR stimulation activates WASp to interact with the Arp2/3 complex (78, 245). However, the interaction between these two proteins can be inhibited by the dephosphorylation of protein tyrosine phosphatase PTP-PEST through proline, serine, threonine phosphatase interacting protein1 (PSTPIP1)-mediated binding to WASp (246). In CD4^+^ T lymphocytes, N-WASP interacts with CaM (71) to activate the Arp2/3 complex in the nucleus, which mediates the Ca^2+^ induced expression of cytokines (247). WAVE2 is another important activator of the Arp2/3 complex. WAVE2 receives signals from the Rac-GTPase as a complex, including Sra-1, Nap1, Abi-1/2, and WAVE2 (248, 249), and is recruited to the membrane (250) for activating the Arp2/3 complex and promoting lamellipodial spreading. Inhibition of WAVE2 expression can lead to impaired IS formation in T cells (251, 252). The acidic region of HS1, NTA, binds to the Arp2/3 complex and contributes to the stabilization of the F-actin branches (253, 254). Tyrosine phosphorylated HS1 was found to bind to the SH2 domain of Vav, stabilizing Vav at the IS (255), which in turn transmits the signal to the Arp2/3 complex.

Related to the above, Formins assist Arp2/3 in regulating the cytoskeleton during T cell activation (256). The Formin mDia 1/3 was shown to regulate the actin kinetics required for recruitment of activated ZAP70 to the IS to promote the phosphorylation of LAT. This suggests an essential role for Formins in early TCR signaling and therefore is important for positive selection of T cells. The downstream TCR signaling involves the interaction of FMNL1 with AHNAK1, which locates FMNL1 to the cell membrane and boosts calcium influx during cell activation (257). Also worth noting is that Inverted Formin2 (INF2), one of the Formins with both actin polymerization and depolymerization activities, is regulated in T cells by Cdc42 and Rac1, which helps MAL-mediated transport of Lck to the plasma membrane and participates in the signaling (258).

A recent study revealed the positive role of coronin in TCR signaling using coronin 1A-deficient T cells. These cells have elevated cAMP levels that activate PKA, leading to defective CREB phosphorylation and suppression of T cell immune responses by inhibiting CaMKK, a signaling molecule downstream of the TCR (259). However, this kind of functional disorder only causes autoimmunity suppression and has no influence on the response to foreign antigen (194).

**Figure 6:**
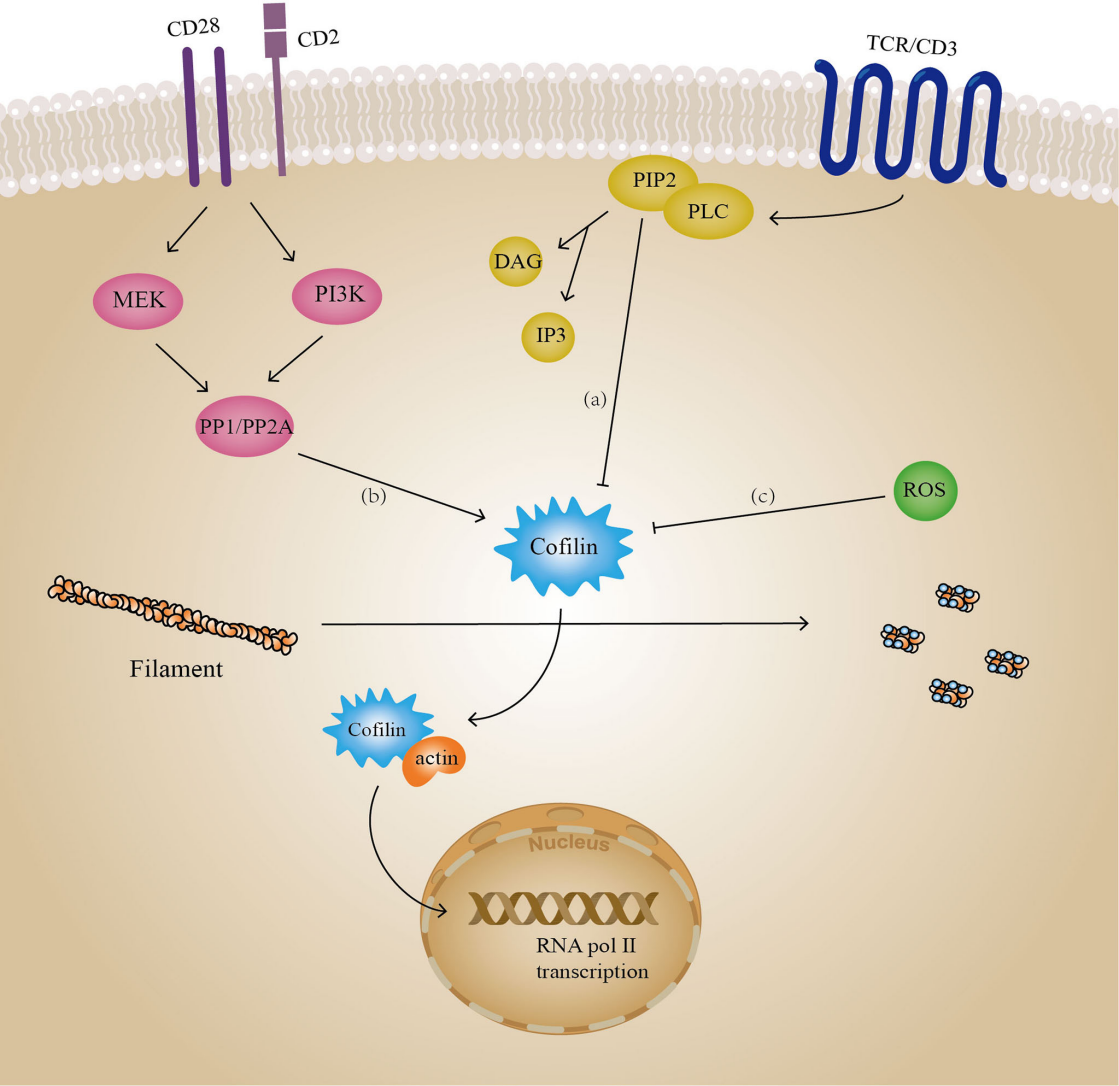
Cofilin involves in T cell signaling. Several signaling pathways in T cells contain cofilin, including: **(A)** PLC-PIP2 pathway belonging to the TCR signaling, **(B)** protein phosphorylation belonging to co-stimulation signaling, and **(C)** protein oxidation. Activated cofilin can mediate actin entry into the nucleus and play a regulatory role in gene transcription. PP1/PP2A: protein serine/threonine phosphatases of type 1 and type 2A, ROS, reactive oxygen species.

Another actin regulator, cofilin, is involved in multiple T-cell signaling pathways, including TCR signaling (**Figure 6**). The cofilin protein sequence contains a phosphatidylinositol binding site which inhibits cofilin activity when bound to phosphatidylinositol (e.g. PIP2) on the plasma membrane, however this effect is independent of cofilin phosphorylation (167, 260). When Phospholipase C (PLC) is activated by TCR signaling as well as amplified signals from CD28 (261, 262), it triggers the decomposition of PIP2, allowing an increase in the amount of active cofilin (262), which may be an early event in T cell activation (263). Furthermore, co-stimulatory signals (e.g. CD40 and CD40L) are also required for T cell activation in addition to the stimulation of TCRs by pMHCs. In the absence of co-stimulatory signals, T cells are in a state of anergy. This is an important mechanism by which the body maintains immunosuppression, and it may also trigger certain pathological responses. It was confirmed that in resting human peripheral blood T lymphocytes (PB-T), dephosphorylation of cofilin occurs after co-stimulatory signals are received by co-receptors (e.g., CD2), rather than activation of TCR/CD3 receptors alone (264, 265). GTPase Ras is a key factor in this process, and dephosphorylation of cofilin requires the synergistic action of both Ras-MEK and Ras-PI3K pathways (266). Under the action of these two pathways, the protein serine/threonine phosphatases of type 1 (PP1) and type 2A (PP2A) bind to cofilin and mediate its dephosphorylation activation (267). It is worth noting that in studies on Jurkat T lymphocyte cell lines, Ras was unable to activate PI3K, reflecting the inconsistency in activation of cofilin by T lymphocytes of different origins (265, 268). Since cofilin contains nuclear localization sequences KKRKK (269), while actin does not, it can mediate actin nuclear ectopic processes upon dephosphorylation (263). This is necessary for the transcriptional activity of RNA polymerase II (270, 271). Additionally, in co-stimulated T cells, dephosphorylated cofilin can regulate the nuclear translocation of NF-κB and promote the production of anti-inflammatory factors, which is one of the mechanisms by which T helper 2 cells (Th2) exert anti-inflammatory effects (272). When cofilin is oxidized and inactivated, it affects costimulatory signaling and leads to T-cell anergy. Cofilin contains four cysteine residues, Cys39, Cys80, Cys140, and Cys148, all of which are potential sites for oxidation. Oxidants such as reactive oxygen species (ROS) may cause the formation of disulfide bonds between Cys39 and Cys80, resulting in the loss of cofilin’s depolymerization activity to F-actin although it can still bind to filaments (273). Moreover, oxidized cofilin tends to undergo mitochondrial translocation, leading to programmed T cell death (274). In contrast, the sensitivity of cofilin to PIP2 is decreased under reducing conditions, resulting in enhanced activity of cofilin (275).

#### 3.1.5 T Cell Cytotoxicity

Upon activation, naïve T cells proliferate and differentiate into different functional subpopulations in response to the local microenvironment and other factors. Some of these cells have killing activity and can differentiate into CTLs, predominantly CD8^+^ CTLs, but also CD4^+^ CTLs have been reported to exist *in vivo* (276). CTLs are able to recognize virally infected and tumorigenic cells and secrete perforin and granzyme to kill these target cells or mediate apoptosis through the death ligands pathway (277–279). Studies have revealed that the function of the Arp2/3 complex is critical for the formation of CTL synaptic protrusions and granule release (280), as well as the CTL’s cytolytic function (281). The importance of Arp2/3 in cytotoxicity is likely due to the actin cytoskeleton maintaining the IS of CTLs. In addition, Arp2/3 is involved in the cycling of TCR, CD8, and GLUT1, which enables the TCR signaling needed for inducing the cytotoxic effects of CTLs on target cells. Also, by binding to Arp2/3, WASp can promote the formation of protrusions and cause deformation of target cells, which was confirmed to enhance perforin and granzyme-mediated killing (280). Furthermore, Formins can also regulate TCR-mediated toxicity by affecting the centrosome polarity of T cells, which is vital for directed release of granules (215).

### 3.2 B Lymphocytes

B cells are important antibody-producing immune cells in humans, the homeostasis of B cells is significant to a well-balanced immunity. Stimulated by antigens, the activation of the BCR initiates the immunological response in B cells. The BCR signaling pathway is regulated by different molecules and their interaction maintains normal B cell function. In addition to BCRs, B cells also have Toll-like receptors (TLRs) that are involved in innate immunity and function to link specific immunity with non-specific immunity. As a component of B cell signal transduction, TLR signaling also contributes to regulation of B cell activity.

#### 3.2.1 BCR/TLR Signaling

**Figure 7:**
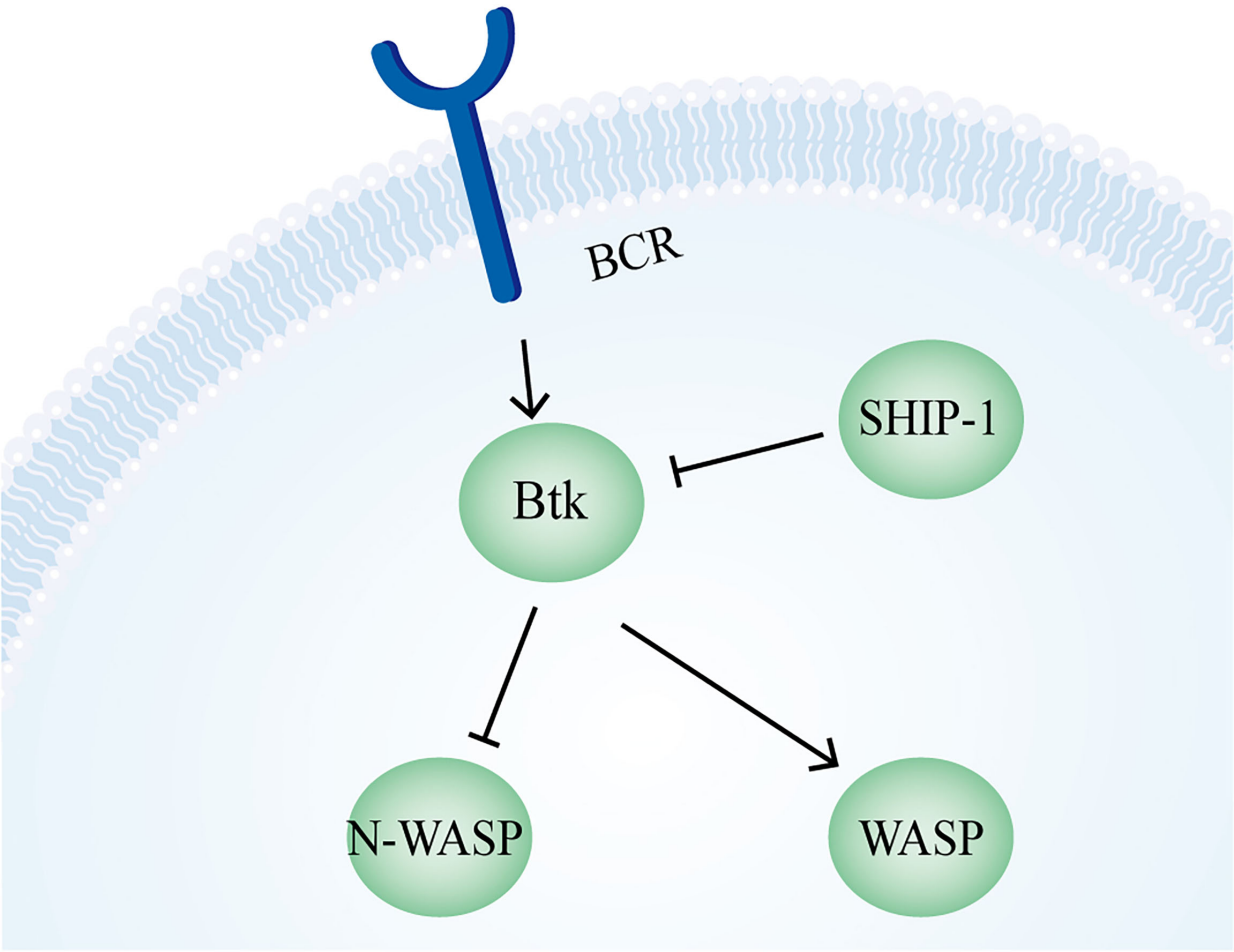
Regulation of N-WASP and WASP in B cells. In B cells, N-WASP is inhibited by Btk, which in turn is negatively regulated by SHIP-1. The regulation of WASP depends on Btk for activation.

In BCR signaling, the unbranched filament formed by the Formin DIAPH1 assists Arp2/3 in the generation of actin foci, which is an important step in the antigen extraction by B cells (282). It was also found that the C-terminus of FHOS, another formin protein widely present in the human spleen, interacts with CD21, which is involved in the regulation of B cell signaling together with the subsequent actin kinetics (283). Upon B cell binding to APCs, the Arp2/3-mediated branched actin network promotes the aggregation of BCR-containing microclusters into a supramolecular activation cluster in the center of the IS, enhancing the BCR signaling (284). The experimental result that the inhibition of the Arp2/3 complex leads to reduced BCR motility also suggests a regulatory role of this complex on B cell signaling (285). N-WASP plays both similar and different roles compared with WASp. On the one hand, in WASp-deficient B cells, N-WASP plays a compensatory role to help BCR clustering and B cell spreading. Also, N-WASP can play a role in BCR microcluster aggregation into central clusters and promote B cell contraction. On the other hand, N-WASP is involved in the up- and down-regulation of BCR signaling. During upregulation, N-WASP and WASp synergistically promote microfilament formation, while N-WASP functions differently from WASP in removing F-actin during cell contraction and signal downregulation. A further study revealed that there is a mutual negative regulatory relationship between WASp and N-WASP. In the BCR signaling pathway, Btk activates WASp but inhibits N-WASP, whereas SHIP-1 can inhibit Btk and activate N-WASP (**Figure 7**) (286).

Ca^2+^ is an important second messenger in BCR signaling, as BCR-induced Ca^2+^ release can act on cofilin *via* the PCLγ and CRAC pathways, and thus regulate B cell adhesion and lamellipodia formation (287). The Ca^2+^ mobilization decreases significantly in coronin1A deficient B cells after BCR stimulation, but the lack of coronin 1A makes no difference for B cell subset development and immune function (288).

**Figure 8:**
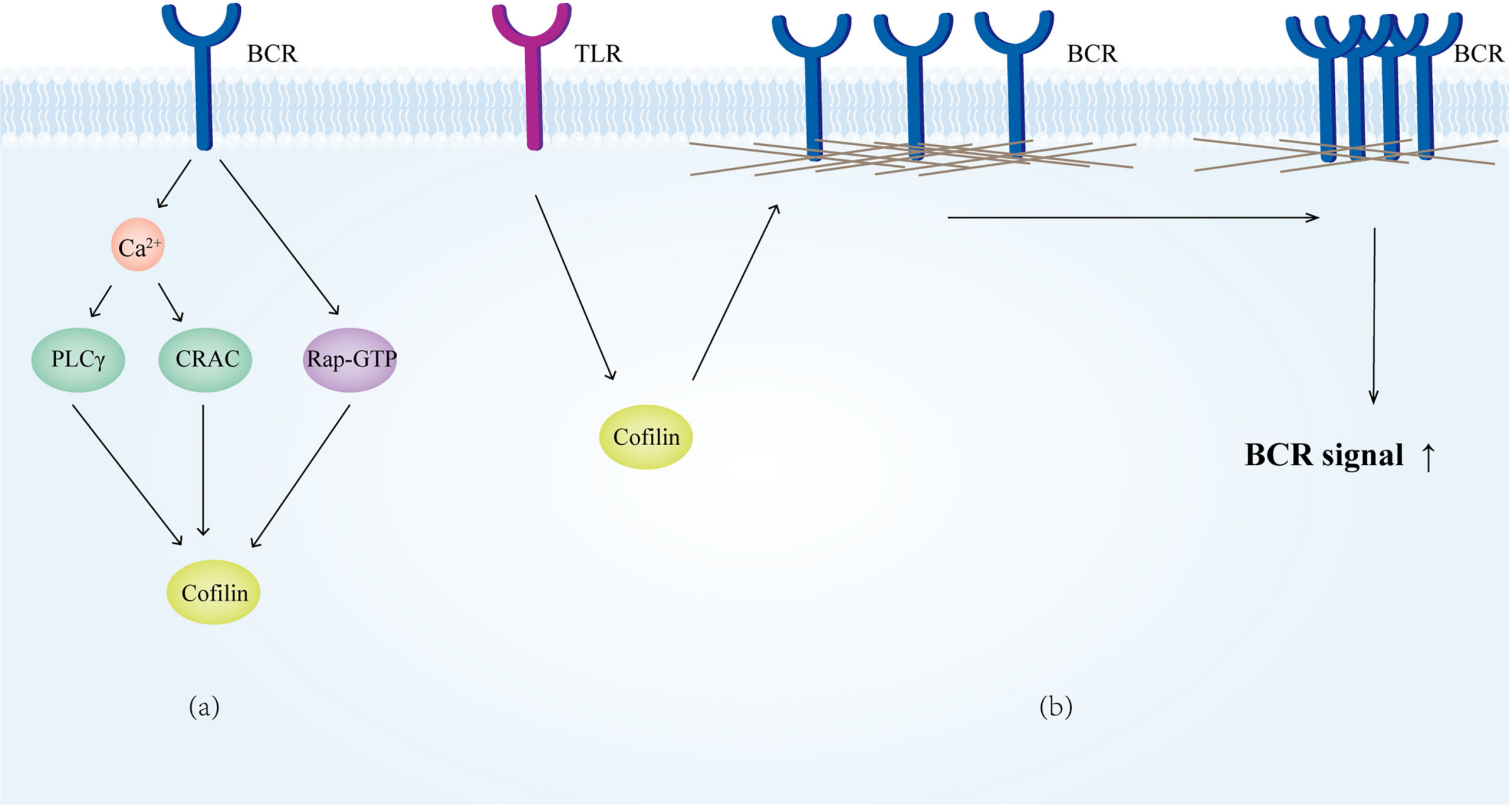
Cofilin in B cells. **(A)** Cofilin receives signals from the BCR. **(B)** Cofilin mediates the depolymerization of microfilaments on the membrane surface upon receiving TLR signaling, which increases BCR motility and facilitates the BCR signaling.

Upon signal stimulation, cofilin is dephosphorylated by BCR *via* active Rap-GTP, severing actin filaments, and thereby increasing the mobility of the BCR and promoting B-cell spreading (289). Meanwhile, the Rap-cofilin pathway promotes the polarization of MTOC toward the APC contacting site, which is an important step in the process of B cell IS formation (290). In addition, cofilin is activated by TLR signaling stimulation, reducing the restriction of the BCR by the actin cytoskeleton and facilitating the signal transduction of the BCR (291) (**Figure 8**).

Recent studies have revealed that WDR1, together with LIMK, an enzyme that phosphorylates cofilin and negatively regulates it, constitute the WDR1-LIMK-cofilin axis, an important mechanism for regulating cSMAC formation and BCR signaling in B cells. This process involves WDR1 and LIMK working together to maintain cofilin at an equivalent activity (neither too high nor too low), thus achieving an optimal level of actin retrograde flow (292).

#### 3.2.2 Others

Many other B cell activities are modified by actin regulators, such as anti-infection, cell development and antibody secretion.

For cell development, the latest studies revealed that the interaction between WASp and other signaling molecules facilitates B cell development and movement. Mst1 and WASp are significant for central and peripheral development of B cells and can adjust mutual localization and function (293). Deficiency of DOCK2 reduces the activation of WASp and accelerates its degradation, causing dysfunction of actin accumulation and affecting the early activation process of B cells (294). Similar to T cells, the reduction of marginal zone B cells in N-WASP conditional knockout mice indicates the promotional effect of this protein on peripheral B cell development. Further studies showed that conditional double knockout WASp and N-WASP exhibited more severe B cell developmental impairment and dysfunction than WASp KO, indicating the positive role of N-WASP in B cell development (72). More severe abnormalities were found in B-cells of WHR1-deficient patients than in T-cells. On the one hand, the number of B-cell precursors is reduced, and more peripheral blood B cells are in an immature stage; on the other hand, this defect leads to abnormal BCR/TLR signaling, generates elongated B-cell synapse, and affects B cell survival (199). Besides intrinsic accommodation of B cell growth, environmental factors such as the extracellular matrix components may influence the motility of B cells (295). The actin dynamics regulated by the Arp2/3 complex gives B cells this ability to exhibit adaptive mobility in different environments (296).

For the response to antigen stimulation, it has been demonstrated that in secondary lymphoid organs, cofilin mediates B cell responses to two common antigens, namely soluble and membrane-associated antigens, in the non-phosphorylated form (297). Differences in the distribution and kinetic characteristics of cofilin under the two antigen inductions explain the different reactivity of B cells to these two antigens (297). N-WASP in B cells is closely linked to autoimmune responses (298). It was found that specific knockdown of N-WASP in B cells resulted in elevated levels of autoantibodies, and mouse B cells lacking N-WASP exhibited higher levels and longer duration of activation. This suggests that N-WASP is a key inhibitor of B cell activation, and this inhibition is essential to terminate the immune response and prevent autoimmunity (286). Relatedly, simultaneous knockdown of WASp and N-WASP in B cells results in more severe proliferation defects in B cells and reduced levels of IgG autoantibodies compared to specific knockdown of the WASp gene, suggesting that N-WASP expression in B cells is required for the development of autoimmunity, which signifies N-WASP as a new therapeutic target to control autoimmunity in WAS patients (299). Analogously to that in T cells, the Arp2/3 complex is important to the formation and activity of the IS in B cells. It was demonstrated that this complex is involved in the formation of dynamic actin structures at the IS of B cells (282). Also, upon B lymphocyte activation, the HS1-dependent Arp2/3 complex is recruited at the IS through Syk-mediated tyrosine phosphorylation (300), which results in a reduction of Arp2/3 complex at the centrosome, leading to its nuclear separation and polarization to the IS (301), a process that maintains the immunomodulatory role of lymphocytes.

Additional benefits WASp provides for lymphocytes includes protection from DNA damage. WASp acts as a novel element to link irradiation-induced DNA damage signaling with the Golgi dispersal response (GDR), which has the ability of ensuring genome stability and contributes to cell survival under DNA damage. The loss of WASp triggers accumulated DNA damage and causes the failure of GDR, resulting in the dysfunction of human T and B lymphocytes (302).

### 3.3 NK Cells

As a vital member of lymphocytes, natural killer cells (NK cell) are not only involved in fighting tumors and infections, but also participate in hypersensitivity and autoimmune disease. Its natural killing activity is independent of antigen stimulation and is not restricted by MHC.

#### 3.3.1 NK Cell Cytotoxicity

NK cells can kill target cells by forming IS and releasing lytic granules (303). The activity of NK cells is regulated by cytoskeletal proteins (304, 305), of which the Arp2/3 complex promotes the aggregation of NK receptors in the lytic synapse, allowing for stable binding between NK cells and target cells. This has been demonstrated in studies showing that the lack of Arp2/3 causes defective cell adhesion to target cells, which inhibits lytic synapse actin assembly, thus affecting the lysis activity of NK cells (306). Additionally, examination at the nanoscale level found that the Arp2/3 complex also plays an active role in lytic granule secretion (307).

Tβ4 enhances the cytotoxicity of NK cells by increasing the expression of intercellular adhesion molecule-1 (ICAM-1) and LFA-1, as well as cytolytic granule exocytosis (308). Furthermore, the expression of Tβ4 is upregulated by IL-18 and in turn, Tβ4 enhances IFN-γ secretion mediated by IL-18 in NK cells, which positively influences the immunomodulatory role of NK cells (309).

With respect to the WASp family, WASp is localized in the IS with F-actin and regulates cytotoxicity functions in NK cells (310, 311). This correlates with the interaction of DOCK8, which regulates the polarization of WASp and mediates the cytotoxicity of NK cells (312). The Y141 tyrosine of WASH can be phosphorylated by the Src family kinase Lck, thus exerting its role in promoting the movement and release of granules and assisting the cytotoxic effects of NK cells (313). Formins also help in NK cell activity. It has been demonstrated that the effect of hDia1 on NK cell toxic effects is actin-independent, instead it mediates the formation of microtubule networks through enrichment at the lytic synapse to facilitate the transport and secretion of lytic granules (306). In addition, the function of hDia1 in the formation of filopodial protrusions makes it a vital factor involved in the migration and adhesion of NK cells (306). What is more, cofilin is phosphorylated in response to LIMK stimulation and regulates the remodeling of the actin cytoskeleton in NK cells, thus affecting its cytotoxicity (314).

Finally, a recent mass spectrometry study of NK cell protein expression in severe aplastic anemia (SAA) patients found that the Arp2/3 complex is downregulated in NK cells (315), suggesting a role in the disease-protective function of NK cells in SAA.

#### 3.3.2 Others

As for cell movement, the knockdown of N-WASP partially rescues the inhibition of NK cell migration towards CXCL12 gradient, suggesting its involvement in the positive regulation of NK cell motility (316). Furthermore, the results of *in vitro* experiments suggest that cofilin in NK-92 cells is stimulated by leptin under physiological conditions, which may promote cell migration and mediate the positive effects of leptin on NK cell morphology in a healthy environment (317).

It has been reported that WASp deficiency affects the activation of NK cells and DCs, as DCs in WAS KO mice have weaker induction of NK cells, thus influencing the immune response.

Up to now, it is believed that coronin has little function in NK cells, although further research is still needed (318).

## 4 Related Diseases

### 4.1 Cancer

Since actin filaments are important to cell motility, migration, adhesion, cell growth and cell division, aberrant functions of actin regulators could lead to abnormal cellular functions and cytokinesis, thus contributing to the development and migration of tumor cells (319). Moreover, aberrant actin regulators can also influence the cytotoxic functions of T cells and NK cells, resulting in tumorigenesis and progression.

Tβ4 is reported to have tumor-promoting functions that enhance the motility of cancerous cells. Myocardin-related transcription factors (MRTF), which are co-activators of serum response factor (SRF), are linked to epithelial-mesenchymal transition (EMT) and tumor metastasis. Studies have found Tβ4 can bind to actin competitively with MRTF, mediating the activation of the latter by TGFβ and regulating the cytoskeleton and the expression of tumor-associated proteins, thus enhancing the motility of tumor cells and leading to tumor progression and metastasis (320). Additionally, in glioma cells, Tβ4 inhibition reduces the migratory capacity and aggressiveness of tumor cells and is a potential target for the treatment of glioblastoma (321).

Many recent studies have focused on the relationship between WASp and cancer. WASp is important for tumor suppression and killing and considered a new possible therapeutic target in cancer. In T cells, constitutive activation of WASp improves cytotoxic clearing of tumor cells (322). Interestingly, studies have shown that WASp plays opposite roles in malignant and benign lymphocytes. In benign T and B cells, WASp is a tumor-suppressor protein, however it acts as a tumor activator in malignant lymphocytes, since the lack of WASp leads to an imbalance of CDC42/MAPK and NF-κB/AP-1 signaling pathways, which is important for the development of cancer (323). But a recent study reported that WASp and WIP are tumor suppressors in T cell lymphoma, because high expression of WASp prevents lymphoma growth (189). Further studies are needed to figure out the role of WASp in malignant lymphocytes. In WASp deficient mice, the antitumor functions of NK cells and DCs are weakened (324). WASp deficient patients have increased granzyme B and degranulation, along with enhanced production of IFN-γ. Additionally, the expression levels of DNAM-1, LAG-3, KLRG1 increase while CD56 expression decreases, indicating a lack of NK cell phenotype. This correlates with defective F-actin accumulation and disruption of lytic synapse formation. All these changes in NK cells lead to a weaker anti-tumor response (322, 325). In addition, inhibiting Cdc42, which activates NKG2D, WASp and N-WASp to regulate NK cell migration, may impact immunoreaction and evasion in tumors (316).

The Arp2/3 complex also shows strong relevance in cancer development. The contribution of the Arp2/3 complex in podosome formation (326, 327) makes it an important factor in enhancing the mobility of cancer cells. N-WASP can promote cancer progression by activating Arp2/3 and affecting the tumor killing function of NK cells. For example, it has been demonstrated that N-WASP exerts actin-regulatory activity in colorectal cancer metastasis (328), while in breast cancer cells, N-WASP enables rapid actin reconstitution, accumulating large amounts of F-actin at NK cell synapses, thus resisting NK cell attack and inducing immune escape (329). Furthermore, a clinical study on esophageal carcinoma showed that overexpressed WASH maintained the stem cell phenotype of cancer cells by promoting IL-8 production, thus promoting the progression of the carcinoma. Additionally, *in vivo* experiments found that the inhibition of WASH expression slowed the progression of tumors, which suggests WASH be a potential target to intervene in human esophageal carcinoma (330).

A study performed on an oral squamous cell carcinoma (SCC) cell line revealed that PI3K signaling-dependent formin, FHOD1, was specifically upregulated during EMT in tumor cells, which promoted morphological changes and enhanced the invasiveness of cancer cells (331). Similarly, an *in vivo* study found that mDia1 promoted the TEM capacity of leukemia cells and played an active role in the progression of leukemia, and conditional inhibition of mDia1 expression might be a latent way to treat leukemia (332). Studies in Molt-3 and Jurkat cells indicate that human formin-2 (FMN2) expression is inhibited by upregulated microRNA-144 (miR-144), affecting normal cellular activities and providing an explanation for the tumor suppression mechanism of miR-144 (333). Additionally, FMNL1 is expressed in a variety of malignant tissues, while an antigenic peptide (FMNL1-PP2) derived from FMNL1 was experimentally shown to induce specific T cells to exert killing effects on tumors, including lymphomas (334).

Cofilin is also closely associated with tumor development. The regulation of cofilin phosphorylation by Myeloid cell leukemia 1 (MCL-1) is associated with tumor cell activity in B-cell lymphoma 2, and the inhibition of this effect of MCL-1 has become important for tumor therapy (335). Moreover, in malignant T lymphoma cells, it was found that blocking the dephosphorylation of cofilin led to apoptosis, which might have a promising role in arresting tumor progression (336). Cofilin’s regulatory role on the cytoskeleton makes it an important molecule in regulating the migration of tumor cells (337). It has been demonstrated that chitinase 3-like 1 (Chi3l1) dysregulation, present in many solid tumors, inhibits the accumulation of phosphorylated cofilin, hence promoting tumor cell metastasis, however, activation of RIG-like helicase can rescue this process (338). Specifically, in breast cancer, various molecules, such as LMO2, regulate the actin cytoskeleton by interacting with cofilin and altering the dynamics of cell adhesion and lamellipodial, thereby promoting the metastasis of cancer cells and increasing their invasiveness (339–341). Additionally, in the tumor microenvironment, granulocytes or macrophages can mediate oxidative stress (342, 343), which may lead to a loss of functional activity of cofilin, and in turn affect the function of T cells. In tumor treatment, heat therapy can inhibit tumor cell migration by phosphorylating cofilin through high temperature, but it should be noted that the inhibitory effect of Hsp70 overexpression on this process can reduce the efficacy (344).

Analysis of the proteome of Pancreatic ductal adenocarcinoma (PDAC) tissues showed that protein level of WDR1 increased with the clinical progression of PDAC. Further studies revealed that WDR1 is associated with tumor cell growth and metastasis and is involved in the deubiquitination of β-Catenin in PDAC cells by interacting with USP7, thereby regulating Wnt/β-Catenin signaling in PDAC cells (345).

Overall, comprehensive understanding of the role of various actin regulators in tumorigenesis and metastasis can provide new ideas for precise therapy. Moreover, the critical role of actin regulators in immune cells can modulate tumor microenvironment and affect the outcome of immunotherapy. Therefore, further studies are required in this field to bring more benefit to patients with cancer.

### 4.2 HIV

Acquired immunodeficiency syndrome (AIDS) is a severe infectious disease caused by Human Immunodeficiency Virus (HIV) infection. Although researchers around the world have made great efforts, no effective drugs have been developed to cure AIDS, and there is also no effective vaccine that can be used for prevention. Several actin regulators are reported to be related to HIV infection and may provide latent therapeutic targets for AIDS.

The role of profilin in resisting HIV infection is controversial. In ADP-Heat Shock Protein (ADP-HSP) immunized macaques, profilin can up-regulate the expression of apolipoprotein B mRNA-editing enzyme-catalytic polypeptide-like 3G (APOBEC3G), and increase the level of IgG against CD4^+^ T cells, which provides a new direction of therapy in HIV infection (346). However, another study has shown that through downregulating drebrin, another actin-binding protein, profilins accumulate and suppress the polymerization of actin in CD4^+^ T cells, which increases the entry of HIV (347).

HIV can cause the over-activation of cofilin (348), leading to a weakened T cell cycle and inhibiting T cell activation (349, 350). *In vitro* studies, however, revealed that the use of anti-human α4β7 integrin antibodies partially restored the motility of CD4^+^ T cells (348). Cofilin and the cytoskeleton have an important role in HIV infection (351). First, HIV can activate cofilin through CXCR4-mediated Gαi-dependent signaling pathways, promoting the depolymerization of cortical actin and facilitating its viral nuclear localization in resting T cells (352). Also, chemokines such as CCL19 and CCL21 can produce a similar reaction to promote the nuclear migration of HIV in memory T cells, which is one of the possible mechanisms for the establishment of HIV latency (353, 354). Furthermore, the migration of infected T cells can be restricted by HIV through the binding of Nef to the cellular kinase Pak2, which phosphorylates cofilin and inhibits actin dynamics (355).

*In vitro* and *in vivo* studies both showed that intracellular Tβ4 expression in T cells and macrophages was reduced after HIV-1 infection, indicating a regulatory effect of Tβ4 in T cell immunity (356).

N-WASP is one of the ways in which HIV-1 infects T cells. The Nef protein of HIV-1 inhibits both the activation of N-WASP and its recruitment in the T cell contact region, which also interferes with the role of its upstream regulatory molecules, Rac1 and Cdc42, in regulating actin remodeling, thus inhibiting the proper TCR signaling pathway (357).

Arp2/3 plays a critical role in HIV infection. Earlier studies have found that during pathogen infection the Arp2/3 complex of host cells can be activated by the vaccinia virus-encoded A36R protein, contributing to virus transmission (358). Also, baculovirus activates the Arp2/3 complex similarly, facilitating viral proliferation (359). The action of HIV on the Arp2/3 complex is an important component in the development of AIDs. Through the Rac1-IRSP53-WAVE2-Arp2/3 signaling pathway, HIV can invade host cells (360) and promote the assembly and release of virus-like particles (VLPs) (361). Lack of the Arp2/3 complex inhibits HIV infection of CD4 T cells, implying it could be a potent target for the treatment of AIDs (362, 363).

Furthermore, Dia1 and Dia2 also play an important role in early HIV-1 infection by helping to form a stable microtubule network and promoting viral uncoating (364). In HIV-1 infected DCs, Dia2 facilitates HIV-1 infection of T cells by regulating actin assembly to assist filopodia formation, while Slit2N was found to inhibit this effect (365, 366).

Determining the relationship between HIV infection and actin regulators will be helpful in developing more effective drugs in the prevention and treatment of AIDS in the future.

### 4.3 Others

Because of the vital role of the cytoskeleton in lymphocytes, actin regulators are also specifically related to other diseases including autoimmune diseases and infectious diseases.

High expression of Tβ4 was detected in patients with pulmonary tuberculosis, which is related to inflammation and angiogenesis mediated by HIF-1α and VEGF and may serve as a potential biomarker for diagnosis (367). Moreover, an analogue of Tβ4 was found to have a restorative effect on T-lymphocyte deficiency in uremic patients and used to rescue the immune function in uremic patients (368).

WAS directly influences the essential role of the functional activity of the Arp2/3 complex mediated by WASp in the human body (62). CID, a disease caused by a homozygous mutation in the gene encoding ARPC1B, is similar to WAS (230). And defects in ARPC4 in the epidermis may trigger Psoriasis-like skin complication epidermis (369), while pachygyria may be caused by cortical neuronal migration disorders due to overactivity of the Arp2/3 complex (370). Also, the decrease in cell migration caused by the inhibition of the Arp2/3 complex by factors such as prostaglandin E2 may be responsible for Hirschsprung disease (371, 372). Recent studies have also identified the significance of this complex in bone and neural tissues, suggesting that defects in Arp2/3 may be associated with the development of intervertebral disc defects (373) as well as Down syndrome and Alzheimer disease (374). In innate immune cells, defective WASp interrupts the autophagy-inflammasome axis (375). And further studies reveal that depending on the interaction with Arp2/3, WASp plays an essential role in the autophagy process, especially during the autophagosome formation and the delivery of autophagosomes to lysosomes (376), which contributes to the development of auto-inflammatory diseases. Additionally, the dysfunction of endogenous inhibitory proteins of Arp2/3 also leads to the development of several diseases. For example, the over-expression of coronin 1A causes the over activation of CD4^+^ T cells and CD8^+^ T cells in aplastic anemia and hemophagocytic syndrome (377).

Formins perform different roles in the development of several diseases. For example, the promotion of CD4^+^ T cell migration by FMNL1 may be one of the pathogenic mechanisms of equine recurrent uveitis (ERU) (378), suggesting its role in autoimmune diseases. In addition, it is noteworthy that in the chemical-induced asthma mouse model, the expression of cofilin in B cells differs from that in normal individuals (379), suggesting a link between cofilin and the progression of B cell-associated diseases.

Another point of interest is that mutations in the WDR1 gene have been found to cause lazy leukocyte syndrome, a disease characterized by impaired neutrophils, recurrent infections, and stomatitis (380–382). In combination with other findings (198, 383), this mutation impairs the regulation of actin by WDR1, leading to increased levels of F-actin, which induces an IL-18-mediated inflammatory response downstream. Thus, WDR1 is extremely important for neutrophil function and its deficiency can lead to auto-inflammation and immunodeficiency (179).

## 5 Conclusions

Here, we discussed a selection of the most representative actin regulators whose functions in universal cells have been studied extensively, therefore we focused on their roles in lymphocytes. Many of the identified actin regulators to date have important roles in the development and functional activity of lymphocytes. The majority of these roles are performed through cytoskeletal modulation by actin, leading to changes in cell structure or polarity that affect lymphocyte development and immune activity. It is also possible that actin regulators act as effector molecules involved in cellular signaling pathways, thereby regulating cellular activities such as gene expression, where actin may not be necessary. It is worth mentioning that these actin regulators do not exist independently. Actin regulators with similar/opposite functions coordinate/antagonize each other intracellularly to form a complete cytoskeletal regulatory network. Meanwhile, these actin regulators are not in complete parallel in terms of their functions, and multiple regulatory effects on lymphocytes need to be realized through interactions between upstream and downstream actin regulators. Therefore, damage or deletion of different actin regulators causes cellular developmental disorders and malfunctions to different degrees, leading to the development of various diseases. Particularly, in immune system diseases, abnormalities of these proteins often lead to immune overload or immunosuppression by affecting the normal signaling of intrinsic immune cells.

Unfortunately, not all of the actin regulators are fully discussed here since little is known about the functional role of some of the recently discovered actin regulators in lymphocytes. In particular, proteins with more specific functions, such as WASH, may have more scope for investigation in lymphocytes. In addition, we noticed that several actin regulators are closely related to AIDS and tumors, and previous studies have suggested the potential value of targeting these proteins for treatment, so a combine study of several related actin regulators could be followed as an entry point to find the most likely therapeutic modality.

## Author Contributions

JS, XZ, and XF wrote the article and drew the figures. BY and CL organized the draft and revised the draft. HM and PL reviewed, proofread, and revised the draft. JS, XZ, and XF contribute equally to this work. All authors contributed to the article and approved the submitted version.

## Funding

This work was supported by National Natural Science Foundation of China (31970839), HUST Academic Frontier Youth Team (2018QYTD10), Independent Innovation Research Fund of Huazhong University of Science and Technology (2020kfyXGYJ017), and National Undergraduate Training Program for Innovation and Entrepreneurship (202110487122).

## Conflict of Interest

The authors declare that the research was conducted in the absence of any commercial or financial relationships that could be construed as a potential conflict of interest.

## Publisher’s Note

All claims expressed in this article are solely those of the authors and do not necessarily represent those of their affiliated organizations, or those of the publisher, the editors and the reviewers. Any product that may be evaluated in this article, or claim that may be made by its manufacturer, is not guaranteed or endorsed by the publisher.
